# Bovine WC1^+^ and WC1^neg^ γδ T Lymphocytes Influence Monocyte Differentiation and Monocyte-Derived Dendritic Cell Maturation during *In Vitro Mycobacterium avium* Subspecies *paratuberculosis* Infection

**DOI:** 10.3389/fimmu.2017.00534

**Published:** 2017-05-22

**Authors:** Monica M. Baquero, Brandon L. Plattner

**Affiliations:** ^1^Department of Pathobiology, Ontario Veterinary College, University of Guelph, Guelph, ON, Canada

**Keywords:** *Mycobacterium avium* subspecies *paratuberculosis*, γδ T lymphocytes, WC1, macrophages, monocytes, dendritic cells, mononuclear phagocyte system

## Abstract

During early *Mycobacterium avium* subspecies *paratuberculosis* (*Map*) infection, complex interactions occur between the bacteria, cells from the mononuclear phagocyte system (MPS) including both resident (macrophages and dendritic cells) and recruited (monocytes) cells, and other mucosal sentinel cells such as γδ T lymphocytes. Though the details of early host–pathogen interactions in cattle remain largely underexplored, our hypothesis is that these significantly influence development of host immunity and ultimate success or failure of the host to respond to *Map* infection. The aims of the present study were to first characterize monocyte-derived MPS cells from young calves with respect to their immunophenotype and function. Then, we set out to investigate the effects of WC1^+^ and WC1^neg^ γδ T lymphocytes on (1) the differentiation of autologous monocytes and (2) the maturation of autologous monocyte-derived dendritic cells (MDDCs). To achieve this, peripheral blood WC1^+^ or WC1^neg^ γδ T lymphocytes were cocultured with either autologous freshly isolated peripheral blood-derived monocytes or autologous immature MDDCs (iMDDCs). We began by measuring several markers of interest on MPS cells. Useful markers to distinguish monocyte-derived macrophages (MDMs) from MDDCs include CD11b, CD163, and CD172a, which are expressed significantly higher on MDMs compared with MDDCs. Function, but not phenotype, was influenced by WC1^neg^ γδ T lymphocytes: viability of *Map* harvested from monocytes differentiated in the presence of WC1^neg^ γδ T lymphocytes (dMonWC1^neg^) was significantly lower compared to MDMs and MDDCs. With respect to DC maturation, we first showed that mature MDDCs (mMDDCs) have significantly higher expression of CD11c, CD80, and CD86 compared with iMDDCs, and the phagocytic capacity of mMDDCs is significantly reduced compared to iMDDCs. We then showed that γδ T lymphocyte subsets induce functional (reduced phagocytosis) but not phenotypic (surface marker expression) iMDDC maturation. These data collectively show that γδ T lymphocytes influence differentiation, maturation, and ultimately the function of monocytes during *Map* infection, which has significant implications on survival of *Map* and success of host defense during early *Map* infection.

## Introduction

The mononuclear phagocyte system (MPS) comprises monocytes, macrophages, dendritic cells (DCs), and their precursors in the bone marrow (1). Myeloid progenitor cells give rise to circulating monocytes which migrate into various tissues where they function as resident tissue macrophages or DCs (2–4). A primary function of cells from the MPS under normal physiologic conditions is to maintain homeostasis in peripheral tissues (5). During inflammatory processes, they play a crucial role initiating and regulating immune responses by processing and presenting antigens to naïve T lymphocytes (6). Cells from the MPS share several surface markers and functions, making it difficult to clearly define the distinction between them (6). The phenotype of monocytes, macrophages, and DCs of humans and mice has been extensively studied and these cells have been classified according to the expression of specific markers [reviewed in Ref. (7)]. Classification of bovine DCs, including monocyte-derived dendritic cells (MDDCs) based on phenotype and function has been described [reviewed in Ref. (8)]; however, little is known about the phenotype and function of bovine monocytes, macrophages, and the expression of phenotypic surface markers after monocyte *in vitro* differentiation.

During initial exposure to pathogens at mucosal surfaces, cells from the MPS including tissue-resident macrophages and DCs interact with other immune cells, such as γδ T lymphocytes at mucosal surfaces. γδ T lymphocytes are considered to be a bridge between innate and adaptive immune systems. In cattle, γδ T lymphocytes are classified broadly as WC1^+^ and WC1^neg^ according to their expression of the workshop cluster 1 (WC1) molecule, which is a transmembrane glycoprotein belonging to the scavenger receptor cysteine-rich family (CD163) (9). WC1^+^ γδ T lymphocytes are considered pro-inflammatory (9) and less is known about the function of WC1^neg^ γδ T lymphocytes; however, it is believed that they are mucosal sentinel cells, given their presence at mucosal surfaces (10). Human and murine γδ T lymphocytes have been the most widely studied. In these species, γδ T lymphocytes recognize pathogen-associated molecular patterns (PAMPs) through pattern-recognition receptors (11), execute their effector functions without clonal expansion because they are not major histocompatibility complex (MHC)-restricted (12, 13), and present antigens to naïve αβ T lymphocytes (14). During adaptive immune responses, γδ T lymphocytes develop memory responses (15, 16), induce DC maturation (17), and polarize into T_H_1-, T_H_2-, T_H_17-, T_FH_-, or T_REG_-effector functions based on the cytokine milieu in which γδ T lymphocytes encounter the antigen (17–20). In cattle, γδ T lymphocytes have shown to produce pro-inflammatory cytokines, such as IFN-γ and IL-17A (18–21), regulate granuloma development (22), have regulatory effects (23, 24), and modulate macrophage-effector functions (25, 26).

This work focuses on studying the specific interactions of bovine γδ T lymphocyte subsets with cells from the MPS in the context of *Mycobacterium avium* subspecies *paratuberculosis* (*Map*) infection *in vitro*. Macrophages and DCs are the primary host cells for *Map* (27, 28), an intracellular bacterium causing paratuberculosis, which is an important mycobacterial infection of ruminants. The disease is characterized by a long subclinical phase (>2 years) (27), followed by a clinical phase in which animals show diarrhea and weight loss caused by inadequate nutrient absorption as a result of progressive granulomatous enteritis (29).

Both γδ T lymphocytes and the MPS play critical roles during the early pathogenesis of *Map* infection in cattle: (1) macrophages are the preferred cell host and the main effector cell during *Map* infection (30); (2) *Map* also infects DCs (28); and (3) monocytes migrate into the intestinal tract during infection and differentiate into effector cells, presumably in the presence of both WC1^+^ and WC1^neg^ γδ T lymphocyte subsets (9, 31). Furthermore, we have previously shown that γδ T lymphocytes influence autologous monocyte-derived macrophage (MDM) effector functions of young calves and heifers during *Map* infection *in vitro* (25, 26). Therefore, the hypothesis for this study was that WC1^+^ and WC1^neg^ γδ T lymphocytes of young calves influence (1) monocyte differentiation and (2) DC maturation during *Map* infection *in vitro*. The specific aims of this study were to first characterize cells from MPS of young calves and then to understand how bovine WC1^+^ or WC1^neg^ γδ T lymphocytes influence autologous monocyte differentiation and DC maturation during *in vitro Map* infection.

## Materials and Methods

### Animals and Blood Collection

All animal procedures in this study were approved by the Institutional Committee on Animal Care at the University of Guelph (Animal Utilization Protocol # 3373). All animals were randomly selected from the Elora Dairy Research Centre, where there is no official paratuberculosis herd certification program; however, the estimated prevalence is near zero in this herd, because it is under continual surveillance for *Map* infection by regular screening for *Map*-specific antibodies using ELISA. No positive antibody tests, clinical or suspect paratuberculosis cases have been diagnosed on this farm for several years. Approximately 120 mL of blood were collected *via* jugular venipuncture using EDTA vacutainer tubes (BD Biosciences, Mississauga, ON, Canada) from seven healthy Holstein calves between 30 and 40 days of age. Number of animals was selected based on sample power calculations. Additional 60 mL of blood from the same calves were collected in serum separator vacutainer tubes (BD Biosciences). Blood samples were stored at 4°C and promptly transferred to the laboratory.

### Peripheral Blood Mononuclear Cells (PBMCs), MDMs, and MDDCs

Under sterile conditions, whole blood was diluted (1:1) with PBS containing 0.5% BSA. PBMCs were isolated from whole blood using Histopaque 1077 (Sigma Aldrich, Oakville, ON, Canada) density gradient centrifugation, counted using a Moxi Z cell counter (Orflo, Hailey, ID, USA), and resuspended in complete medium RPMI 1640 containing 2 mM of l-glutamine and 25 mM of HEPES (Gibco, Carlsbad, CA, USA) supplemented with 5 × 10^−5^ M 2-mercaptoethanol (Sigma Aldrich, Oakville, ON, Canada), with penicillin (1,000 U/mL), streptomycin sulfate (10 mg/mL), and amphotericin B (0.25 µg/mL) (Sigma Aldrich). Our source of serum was 10% autologous serum based on our previous findings that show that cells are not self-reactive to cytokines or other soluble mediators present in autologous serum (26). Cell suspensions were transferred to 175 cm^2^ flasks (Corning, Tewksbury, MA, USA) at a concentration of 7.5 × 10^6^/mL per flask. After 1 h of incubation at 37°C in 5% CO_2_, non-adherent cells were collected by washing each flask 3× with PBS prior to lymphocyte staining and sorting. Adherent cells (monocytes) were detached from the flasks using TrypLE Express (Gibco), washed, counted, and resuspended in complete RPMI. 2 × 10^5^ monocytes/well were cultured in 24-well flat-bottomed plates (Corning). To obtain MDMs, monocytes were incubated for 6 days in complete RPMI. To obtain iMDDCs, complete RPMI was supplemented with 200 ng/mL of recombinant bovine interleukin-4 (Kingfisher Biotech, MN, USA) and 100 ng/mL of recombinant bovine GM-CSF (Kingfisher Biotech). After 6 days of differentiation, cells were used in coculture assays as iMDDCs; some wells of iMDDCs were induced to maturity (mMDDCs) by adding 1 µL/mL of *Escherichia coli* LPS (Sigma Aldrich) to the cell culture for 48 h prior to use in coculture assays as previously described (32, 33).

### γδ T Lymphocyte Sorting

Non-adherent cells collected from the original flasks were resuspended in PBS containing 0.5% BSA, incubated in the dark at 4°C for 15 min with WC1 γδ T lymphocyte monoclonal antibody (BAQ4A, N2 epitope, IgG1, Monoclonal Antibody Center, Washington State University) and γδ T cell receptor monoclonal antibody (GB21A, TCR1-N24 δ chain-specific, IgG2b, Washington State University), washed, and incubated in the dark at 4°C for 15 min with the secondary antibody PE-Cy7 (IgG1, Biolegend, San Diego, CA, USA) and DyLight 405 (IgG2b, Jackson ImmunoResearch, Suffolk, UK). After washing, stained cells were sorted by FACS (Aria IIu, BD Biosciences, Mississauga, ON, Canada). After sorting WC1^+^ and WC1^neg^ γδ T cell populations, cells were resuspended in complete RPMI. The purity of each subset was verified by FACS to be 85–95% and confirmation of a viability over 85% was assessed using the trypan blue exclusion assay described previously (34).

### Cocultures

**Figure 1:**
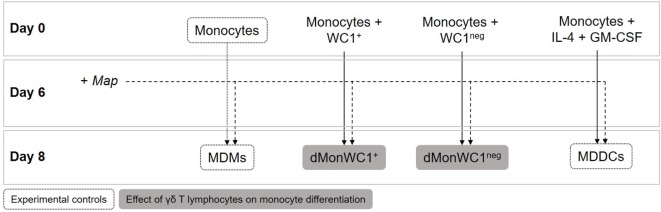
**Monocyte differentiation assays**. 1 × 10^6^ WC1^+^ or WC1^neg^ γδ T lymphocytes were added directly to wells containing 2 × 10^5^ monocytes the same day of peripheral blood mononuclear cell isolation (day 0). After 6 days of differentiation, cultures of monocyte-derived macrophages (MDMs), monocyte-derived dendritic cells (MDDCs), monocytes differentiated in presence of WC1^+^ (dMonWC1^+^) or WC1^neg^ (dMonWC1^neg^) γδ T lymphocytes were obtained. On day 6, live *Map* was added at a multiplicity of infection of 10:1 to evaluate how it affected phenotype of MDMs, dMonWC1^+^ and dMonWC1^neg^; and MDDC after 48 h.

For monocyte differentiation assays, 1 × 10^6^ sorted WC1^+^ or WC1^neg^ γδ T lymphocytes were added directly to wells containing 2 × 10^5^ monocytes the same day of PBMC isolation (day 0). After 6 days, cultures of MDMs, iMDDC, monocytes differentiated in presence of WC1^+^ (dMonWC1^+^), or WC1^neg^ (dMonWC1^neg^) γδ T lymphocytes were obtained (Figure 1). For MDDC maturation assays, 1 × 10^6^ sorted WC1^+^ or WC1^neg^ γδ T lymphocytes were added to wells containing 2 × 10^5^ iMDDCs (day 6) for 48 h (iMDDC + WC1^+^ and iMDDC + WC1^neg^) (Figure 4).

### Infection with *Map*

For monocyte differentiation assays on day six MDMs, iMDDC, dMonWC1^+^, and dMonWC1^neg^ were infected. For MDDC maturation assays, γδ T lymphocyte subsets and bacterial suspensions of *Map* were added on day 6 to iMDDCs. Cell cultures were infected at a multiplicity of infection (MOI) of 10:1 for 48 h with an Ontario-derived clinical bovine *Map* strain (gc86). The *Map* strain was cultured in Middlebrook 7H9 broth supplemented with 10% OADC (oleic acid, albumin, dextrose, catalase) enrichment (BD Biosciences), 0.05% Tween 80 (Sigma Aldrich), and 2 mg/L of mycobactin J (Allied Monitor, Inc., Fayette, MO, USA) referred to below as 7H9-OADC-MJ-T. Optical density (OD) was measured with a spectrophotometer (Genesys 10S VIS, ThermoFisher Scientific, Waltham, MA, USA) at 540 nm wavelength and quantification of bacteria was performed using a standard growth curve. The bacterial suspension was briefly sonicated with a sonic dismembrator (Model 120, Fisher Scientific) at 60% amplitude during 2 s pulses to disperse bacterial clumps. Aliquots of *Map* with viability of 97.4% measured by fluorescein diacetate (Sigma Aldrich) as described previously (25) were kept at −80°C in saline to ensure that the same *Map* passage was used throughout this study.

### Antibodies and Flow Cytometry

Antibodies used in this study are shown in Table 1. Cells were collected 48 h after *Map* infection into serum-free media before staining and assessment (FACSAria IIu, BD Biosciences). Viability was assessed using Zombie NIR fixable viability kit (Biolegend, CA, USA). The acquisition software used was FACS Diva II, and data were analyzed using FlowJo software (Treestar, Inc., San Carlos, CA, USA) (Figure S2 in Supplementary Material shows gating strategy).

**Table 1:** **Anti-bovine monoclonal antibodies used for peripheral blood mononuclear cell immunophenotyping and coculture experiments**.

Primary mAb	Clone	Isotype	Source	Secondary antibody	Source
**Monocyte differentiation**					
CD11c	BAQ153A	IgM	WSU^a^	PE/Cy7	Biolegend
CD163	LND68A	IgG1	WSU^a^	Pacific Orange	TFS^b^
CD11b	MM10A	IgG2b	WSU^a^	DyLight405	JIR^c^
CD14	CAM36A	IgG1	WSU^a^	AF647^d^	TFS^b^
R-PE-conjugated anti-CD1b	CC20	IgG2a	BioRad	–	–
R-PE/Cy5-conjugated anti-CD172a	CC149	IgG2b	BioRad	–	–
FITC-conjugated anti-CD205	CC98	IgG2b	BioRad	–	–
**Monocyte-derived dendritic cell maturation**					
Major histocompatibility complex (MHC)-I	B5C	IgG2b	WSU^a^	AF594	
MHC-II	H42A	IgG2a	WSU^a^	AF647	
R-PE-conjugated anti-CD86	ILA190	IgG1	LSBio	–	–
FITC-conjugated anti-CD80	ILA159	IgG1	BioRad	–	–

*^a^Washington State University, Monoclonal Antibody Center*.

*^b^ThermoFisher Scientific*.

*^c^Jackson ImmunoResearch*.

*^d^Zenon antibody labeling kit*.

### DQ-Ovalbumin (DQ-OVA) Endocytosis Assay

DQ-ovalbumin was added at a concentration of 10 µg/mL to 60 µL of cell suspension (2 × 10^5^ cells) of monocytes, MDMs, iMDDCs, mMDDCs, dMonWC1^+^, dMonWC1^neg^, iMDDC + WC1^+^, or iMDDC + WC1^neg^. Cells were incubated at 37°C for 45 min. After incubation, cells were washed with cold PBS and immediately analyzed by flow cytometry (FACSAria IIu, BD Biosciences) to measure the bright green fluorescence exhibited by the ovalbumin labeled with the pH-insensitive fluorescent dye, boron-dipyrromethene, upon proteolytic degradation after phagocytosis.

### *Map* Viability

Forty-eight hours after *Map* infection of MDMs, iMDDCs, dMonWC1^+^, or dMonWC1^neg^, culture supernatants were collected and wells containing *Map*-infected cells were washed twice with warm PBS to remove free *Map*. Cells were then detached from the flasks using TrypLE Express (Gibco), centrifuged at 400 × *g* for 2 min, and resuspended in sterile saline solution. Cell suspensions were stored at −80°C until further analysis. After thawing, cell suspensions were vortexed vigorously for 10 s to lyse cells, centrifuged at 400 × *g* for 2 min, resuspended in 7H9-OADC-MJ-T, and incubated in 24-well plates for 24 h at 37°C in 5% CO_2_. Contents of each well were centrifuged at 400 × *g* for 10 min and pellets were resuspended in 100 µL of saline solution in 5 mL conical tubes. One microliter of fluorescein diacetate at a concentration of 2 mg/mL (Sigma Aldrich) was added to each tube. After 30 min of incubation at 37°C, samples were analyzed by flow cytometry (Accuri C6, BD Biosciences). Standardization of the procedure and determination of gates were performed using a standard curve generated from known proportions of live and heat-killed *Map* as described previously (25).

### Statistical Analysis

Statistical comparisons were performed using analysis of variance with SAS 9.4 software (SAS Institute, Cary, NC, USA) and GraphPad Prism 6.0 (GraphPad Software, La Jolla, CA, USA). The mean and SEM were calculated in experiments containing multiple data points. A *P* value of ≤0.05 was considered statistically significant.

## Results

### Phenotypic and Functional Characterization of Bovine Cells from the MPS Show Clear Distinctions between MDMs and MDDCs

**Figure 2:**
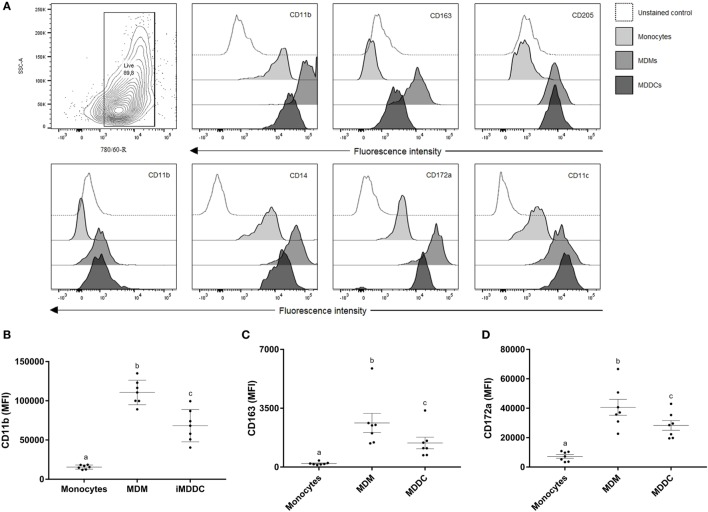
**Surface marker expression of cells from the MPS is heterogeneous**. **(A)** Contour plot of live cell population and flow cytometry analysis of surface expression of CD11b, CD163, CD205, CD1b, CD14, CD172a, and CD11c on monocytes, monocyte-derived macrophages (MDMs), and monocyte-derived dendritic cells (MDDCs). CD11b, CD163, and CD172a are useful surface markers to distinguish MDMs from MDDCs **(B–D)**. Useful markers that distinguish MDMs from MDDCs include CD11b, CD163, and CD172a. Histograms of a representative animal are shown (*n* = 7). Different letters indicate statistically significant difference (*p* < 0.05).

**Figure 3:**
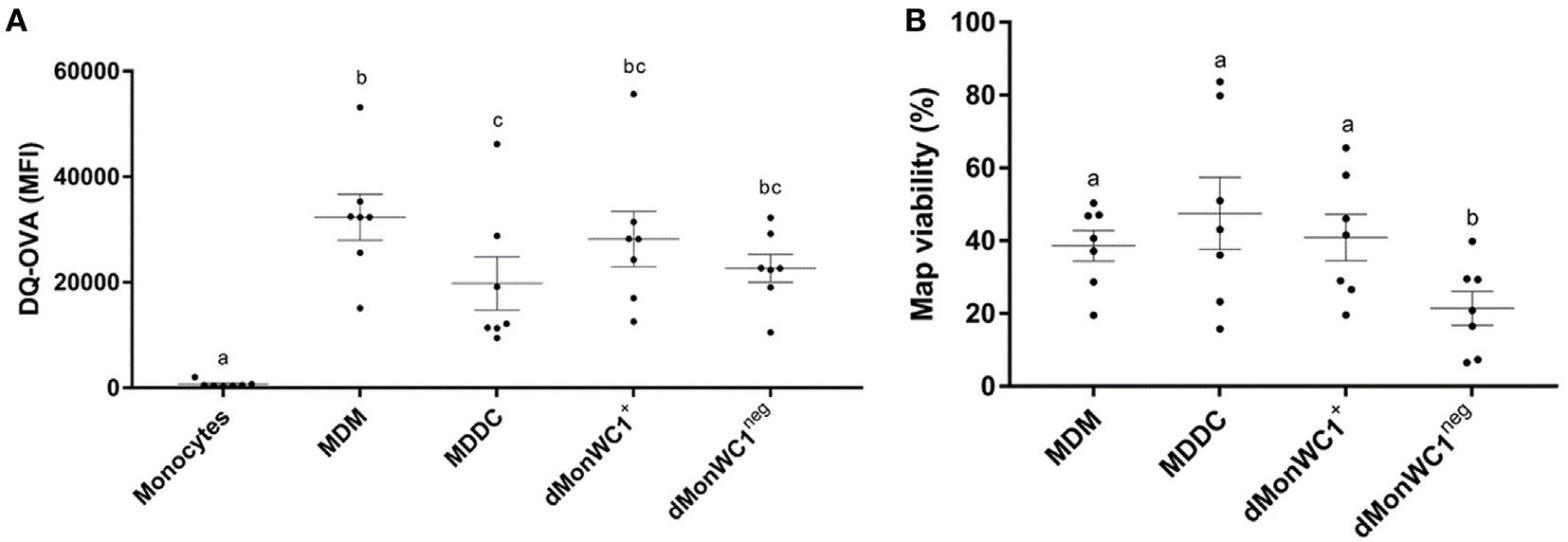
**Cells from the MPS have a heterogeneous phagocytic capacity and the presence of WC1^neg^ γδ T lymphocytes increase the ability of differentiated monocytes to alter *Mycobacterium avium* subspecies *paratuberculosis* (*Map*) viability**. **(A)** Monocyte-derived macrophages (MDMs) had the highest phagocytic capacity, followed by monocyte-derived dendritic cell (MDDC). dMonWC1^+^ and dMonWC1^neg^ showed an intermediate phagocytic activity between MDMs and MDDC. **(B)** *Map* viability measured by flow cytometry after 48 h of infection of MDMs, MDDC, dMonWC1^+^, and dMonWC1^neg^. The viability of *Map* harvested from dMonWC1^neg^ was significantly lower compared to MDMs, MDDC, and dMonWC1^+^. Results are individual animal data points and means ± SEM of seven animals. Different letters indicate statistically significant difference (*p* < 0.05).

To determine the expression of MPS cell markers in this study, several cell types were defined: monocytes were freshly isolated by adherence; MDMs were collected from flasks after fresh adherent monocytes were cultured for 6 days; MDDCs were generated by adding IL-4 and GM-CSF to fresh adherent monocyte cultures (Figure 1). Monocytes display significantly lower expression (*p* < 0.0001) of CD14, CD11b, CD11c, and CD172a compared with MDMs or MDDCs (Figure 2). Fresh monocytes lack expression of CD163, CD1b, and CD205. The markers that help to differentiate MDMs from MDDCs in our system are CD11b, CD163, and CD172a, which are each significantly higher on MDMs compared to MDDCs with *p* values of <0.0001, <0.0001, and 0.0333, respectively (Figures 2B–D). To establish the phagocytic capacity of MPS cells in our study, monocytes, MDMs, and MDDCs were assessed with the DQ-OVA endocytosis assay. As expected, our data show that MDMs had the highest phagocytic capacity followed by MDDC (*p* = 0.0418), while monocytes have minimal phagocytic activity compared with MDMs and MDDCs (*p* < 0.0001 and 0.0028, respectively) (Figure 3A). To compare the ability of MDMs and MDDCs to alter *Map* viability, the viability of *Map* recovered from MDMs and MDDCs 48 h after *in vitro* infection with live *Map* was evaluated by flow cytometry. No significant differences were found between the viability of *Map* harvested from MDMs and MDDCs (*p* = 0.2929) (Figure 3B).

### The Presence of γδ T Lymphocytes during Monocyte Differentiation Does Not Affect Phenotype or Phagocytic Capacity; However, Viability of *Map* Recovered from dMonWC1^neg^ Was Significantly Lower

To analyze the effect of WC1^+^ or WC1^neg^ γδ T lymphocyte subsets on monocytes during differentiation, sorted WC1^+^ or WC1^neg^ γδ T lymphocytes were added to freshly isolated autologous monocytes, and the cells were left in direct contact for 6 days prior to assessment of MPS surface-marker expression (Figure 1). Our data show that the phenotype of monocytes differentiated after 6 days in presence of either WC1^+^ or WC1^neg^ γδ T lymphocytes (dMonWC1^+^ and dMonWC1^neg^) was not significantly different from the phenotype of monocytes differentiated without γδ T lymphocytes (MDMs) in our system. The mean and SD median fluorescence intensity (MFI) of surface markers of MPS in this study are shown in Table S1 in Supplementary Material. The phagocytic capacity of dMonWC1^+^ and dMonWC1^neg^ was assessed with the DQ-OVA endocytosis assay. dMonWC1^+^ and dMonWC1^neg^ showed an intermediate phagocytic activity between MDMs (*p* = 0.5403 and 0.1582, respectively) and MDDC (*p* = 0.3165 and 0.8772, respectively) (Figure 3A). To determine the ability of dMonWC1^+^ and dMonWC1^neg^ to alter *Map* viability, the viability of *Map* recovered from these cells 48 h after live *Map* infection was evaluated by flow cytometry. The viability of *Map* harvested from dMonWC1^neg^ was significantly lower compared with the viability of *Map* harvested from either MDMs (*p* = 0.0492), MDDC (*p* = 0.0044), or dMonWC1^+^ (*p* = 0.0277) (Figure 3B).

### Presence of Live *Map* Does Not Alter Phenotype or Functions of Cells from the MPS

To determine the effect of the presence of *Map* on the phenotype of MPS cells in this system, *Map* was added to cultures of MDMs, MDDC, dMonWC1^+^, and dMonWC1^neg^ for 48 h (Figure 1). The effect of the presence of *Map* was examined by comparing the same cell type (i.e., uninfected MDMs vs. *Map-*infected MDMs). Our data show that infection of MDMs, MDDCs, dMonWC1^+^, or dMonWC1^neg^ with *Map* did not significantly alter surface expression of CD163, CD1b, CD205, CD14, CD172a, CD11b, or CD11c at 48 h post infection (Table S1 in Supplementary Material).

### MDDC Maturation Is Characterized by Upregulation of Surface Expression of MHC-I, CD80, and CD86, and Significant Reduction in Phagocytic Capacity

**Figure 4:**
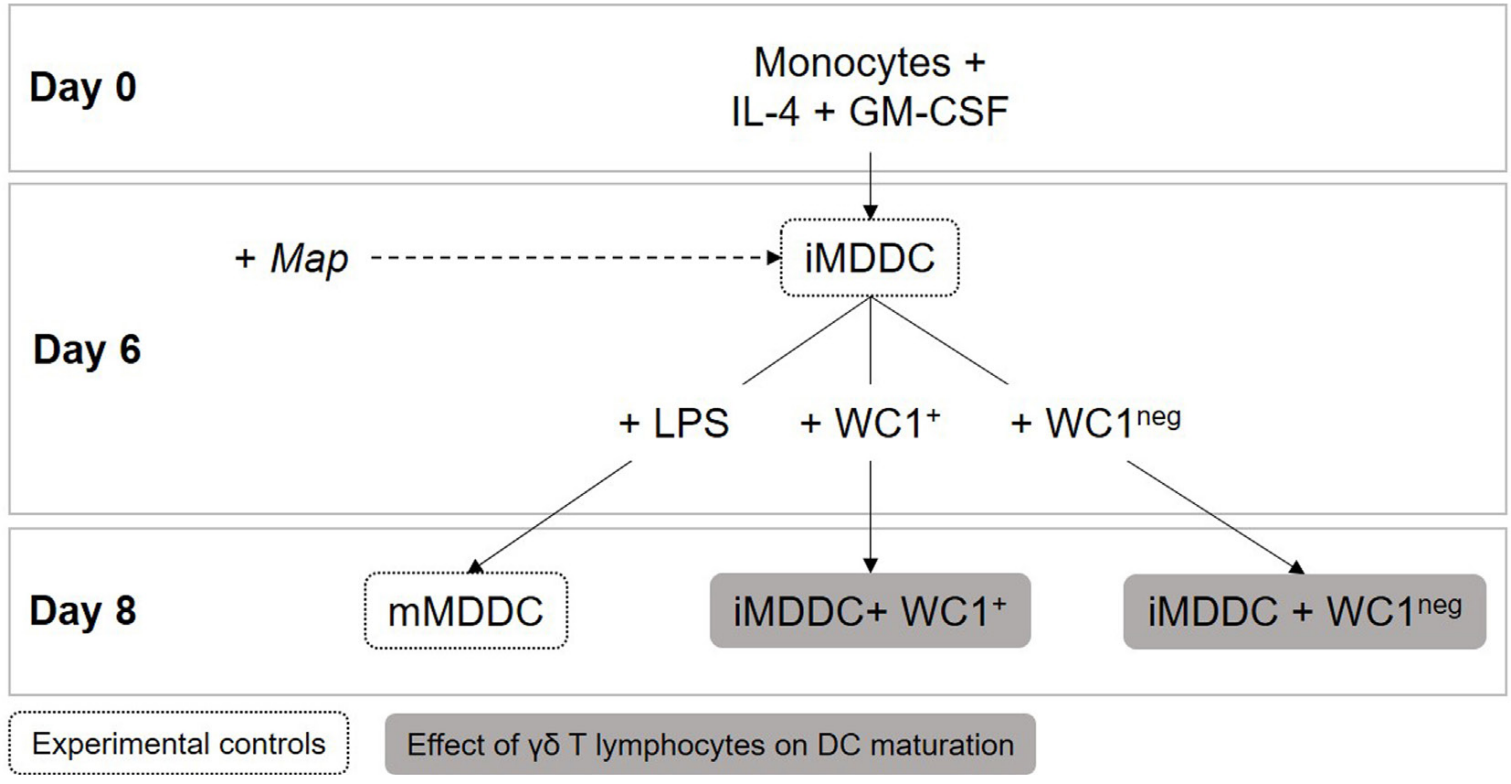
**Dendritic cell maturation assays**. Immature monocyte-derived dendritic cells (iMDDCs) were generated by adding IL-4 and GM-CSF to fresh monocyte cultures. After 6 days of differentiation, 1 × 10^6^ sorted γδ T lymphocytes were added to wells containing 2 × 10^5^ iMDDCs for 48 h (iMDDC + WC1^+^ and iMDDC + WC1^neg^). On day 6, live *Mycobacterium avium* subspecies *paratuberculosis* was added at a multiplicity of infection of 10:1 to evaluate if it affected iMDDC maturation after 48 h.

**Figure 5:**
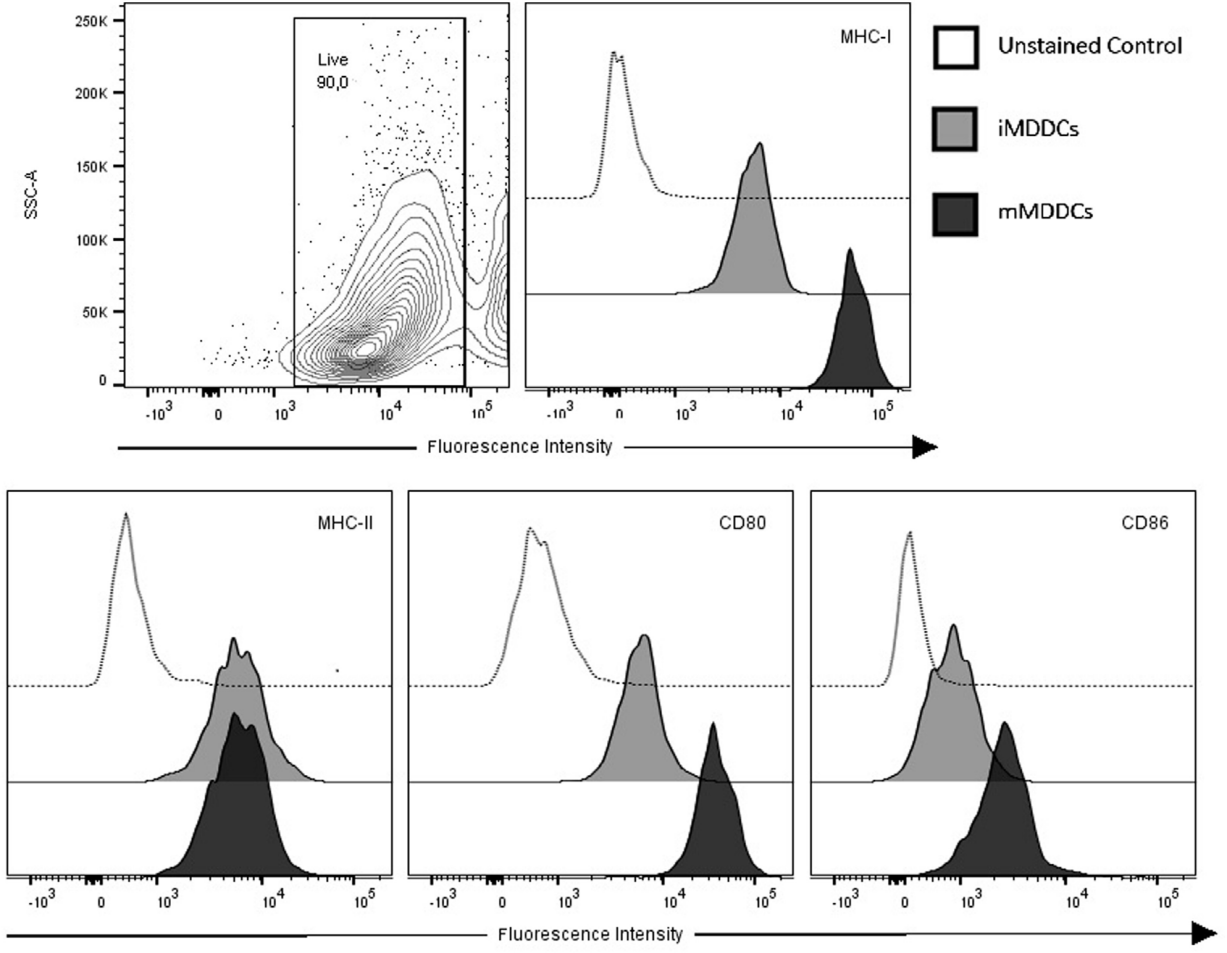
**Monocyte-derived dendritic cells (MDDCs) upregulate surface expression of major histocompatibility complex (MHC)-I, CD80, and CD86 during maturation**. Contour plot of live cell population and flow cytometry analysis of surface expressions of MHC-I, MHC-II, CD80, and CD86 on immature MDDCs and mature MDDCs. Histograms of a representative animal are shown (*n* = 7).

**Figure 6:**
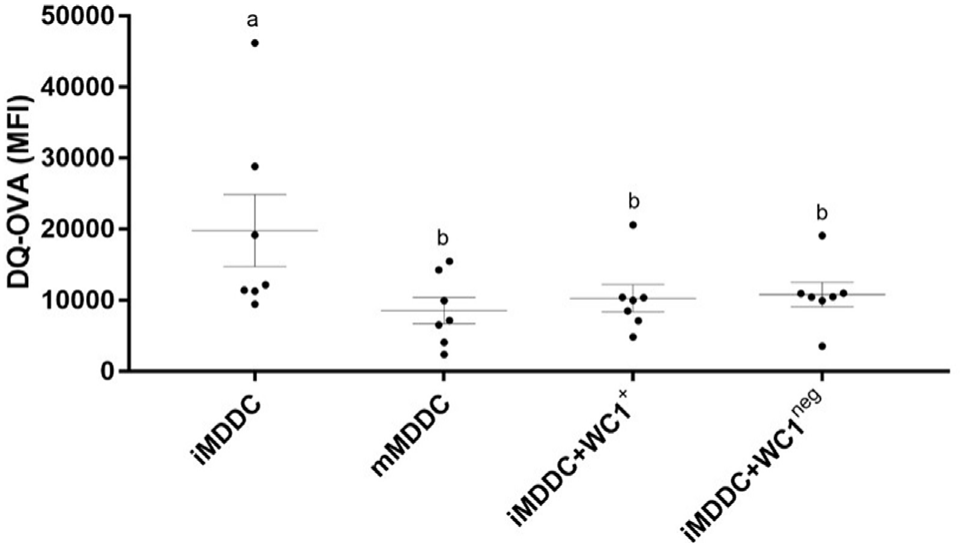
**γδ T lymphocytes induce monocyte-derived dendritic cell (MDDC) maturation**. DQ-ovalbumin endocytosis assay on immature MDDC (iMDDC), mature MDDC (mMDDC), iMDDC + WC1^+^, and iMDDC + WC1^neg^. iMDDC + WC1^+^ and iMDDC + WC1^neg^ had a reduction in their phagocytic ability in the same proportion as mMDDC. Results are individual animal data points and means ± SEM of seven animals. Different letters indicate statistically significant difference (*p* < 0.05).

To examine the DC maturation process, iMDDCs were generated by adding IL-4 and GM-CSF to fresh peripheral blood-derived adherent monocyte cultures and their maturation was then induced by adding LPS for 48 h (Figure 4). As expected, our data showed that expression of MHC-I, CD80, and CD86 were significantly higher on mMDDCs compared with iMMDCs (Figure 5, *p* = 0.0105, <0.0001, and 0.0002, respectively); however, expression of MHC-II on mMDDCs was not significantly different from iMDDCs (*p* = 0.0640) (Figure 5). To study the effect of maturation of MDDCs on their phagocytic capacity, a DQ-OVA phagocytosis assay was performed on iMDDCs and mMDDCs using flow cytometry. Our data showed that the phagocytic capacity of mMDDCs was significantly reduced compared with iMDDCs (*p* ≤ 0.0001, Figure 6).

### WC1^neg^ γδ T Lymphocytes Increase MHC-II Expression on iMDDCs and Both γδ T Lymphocyte Subsets Reduce Phagocytic Capacity of iMDDCs

**Figure 7:**
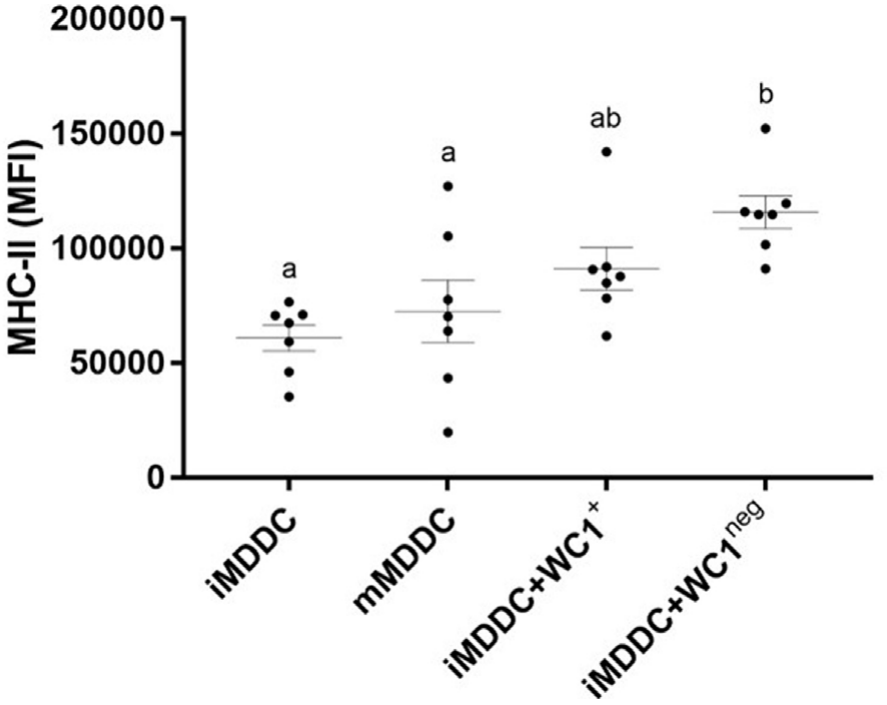
**WC1^neg^ γδ T lymphocytes increase the antigen presenting ability of immature monocyte-derived dendritic cells (iMDDCs)**. Median fluorescence intensity of major histocompatibility complex-II on iMDDCs, mature MDDCs, iMDDCs + WC1^+^, and iMDDCs + WC1^neg^. Results are individual animal data points and means ± SEM of seven animals. Different letters indicate statistically significant difference (*p* < 0.05).

To determine the effect of WC1^+^ and WC1^neg^ γδ T lymphocytes on maturation of MDDCs, sorted γδ T lymphocyte subsets were added to iMDDCs for 48 h (Figure 4). iMDDC + WC1^neg^ had significantly increased expression of MHC-II compared with both iMDDC (*p* = 0.0013) and mMDDC (*p* = 0.0046) (Figure 7). Presence of γδ T lymphocytes did not affect the expression of CD80, CD86, and MHC-I on mMDDCs at 48 h post *Map* infection (data not shown). An interesting finding was increased individual variation of expression of MHC-II on mMDDCs compared with all iMDDCs, iMDDC + WC1^+^, and iMDDC + WC1^neg^, which may suggest that the DC maturation process varies widely between animals. To study the effects of WC1^+^ and WC1^neg^ γδ T lymphocytes on the maturation of MDDCs, γδ T lymphocytes were added to cultures of iMDDC for 48 h and then the DQ-OVA phagocytosis assay was performed using flow cytometry to measure changes in phagocytic ability. iMDDCs cultured in the presence of either WC1^+^ or WC1^neg^ γδ T lymphocytes had significantly reduced phagocytic ability compared to iMDDCs cultured without γδ T lymphocytes (*p* = 0.0111 and 0.0174, respectively) (Figure 6). These findings indicate that both γδ T lymphocyte subsets reduce phagocytosis by iMDDCs in our model.

### Live *Map* Was Associated with Significantly Increased Expression of MHC-I on iMDDC + WC1^neg^

**Figure 8:**
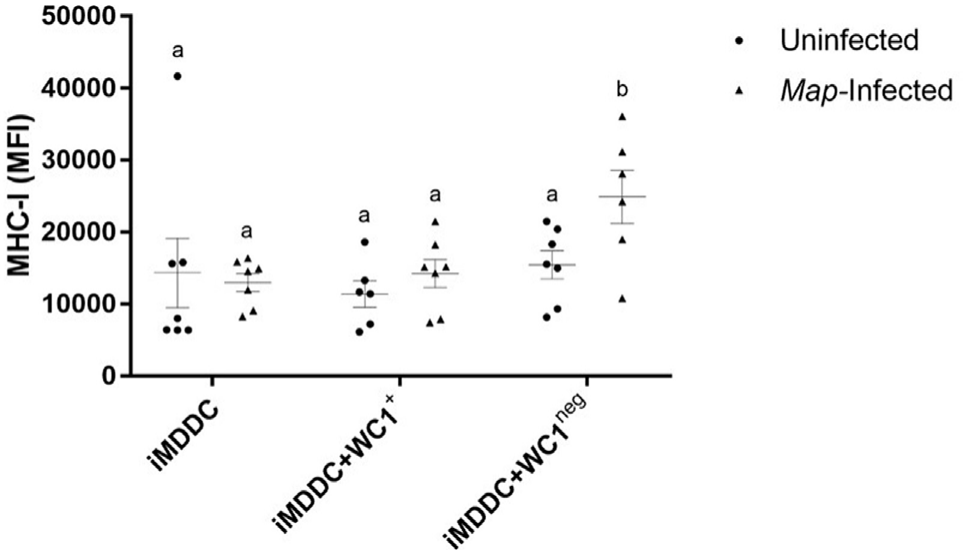
**Live *Mycobacterium avium* subspecies *paratuberculosis* (*Map*) was associated with significantly increased expression of major histocompatibility complex (MHC)-I on immature monocyte-derived dendritic cell (iMDDC) + WC1^neg^**. Infection with live *Map* was associated with significantly increased expression of MHC-I on iMDDC + WC1^neg^. Median fluorescence intensity of MHC-I on iMDDCs, iMDDCs + WC1^+^, and iMDDCs + WC1^neg^. Results are individual animal data points and means ± SEM of seven animals. Different letters indicate statistically significant difference (*p* < 0.05).

To determine the effect of *Map* on maturation of MDDCs, live *Map* was added to cultures of iMDDC, iMDDC + WC1^+^, and iMDDC + WC1^neg^ for 48 h (Figure 4). The presence of live *Map* was associated with significantly increased expression of MHC-I on iMDDC + WC1^neg^ compared to iMDDC + WC1^neg^ unexposed to *Map* (*p* = 0.0090) (Figure 8). The presence of live *Map* did not affect the expression of CD80, CD86, and MHC-II on MDDCs at 48 h post *Map* infection (data not shown).

## Discussion

During the development of host responses against pathogens, monocytes are recruited to the site of infection where they differentiate into effector cells amidst crosstalk with resident tissue immune cells (35, 36). After encountering an antigen, immature DCs begin their maturation process, migrate to local draining lymph nodes where they present antigen to naïve T lymphocytes to initiate, or perpetuate antigen-specific immune responses (37, 38). γδ T lymphocytes are resident sentinel cells in a variety of mucosal surfaces but especially in the ileum, where infection with *Map* is generally assumed to initially occur (39). In this study, we sought to first define and characterize MPS cells from young calves; using that information, we then set out to determine how WC1^+^ and WC1^neg^ γδ T lymphocytes affect (1) monocyte differentiation and (2) DC maturation, both processes important to initiation and propagation of effective immune responses during infection by *Map* and other pathogens. For monocyte differentiation experiments, sorted peripheral blood derived WC1^+^ or WC1^neg^ γδ T lymphocytes were cocultured with freshly isolated autologous monocytes for 6 days so that their phenotype and function could be compared with the experimental controls previously defined: monocytes, MDMs, and MDDCs. For DC maturation experiments, sorted peripheral blood WC1^+^ or WC1^neg^ γδ T lymphocytes were cocultured for 48 h with iMDDCs and then compared with iMDDCs (without γδ T lymphocytes) and mMDDCs (obtained after stimulation of iMDDCs with LPS for 48 h).

Cells from the MPS share precursors as well as several surface markers and functions which makes it difficult to clearly distinguish between them (6). We show that freshly isolated peripheral blood monocytes express low levels of CD14, CD11b, CD11c, and CD172a and lack expression of CD163, CD1b, and CD205. These data are consistent with a recent review showing that bovine monocytes express CD172a but lack expression of CD1b and CD205 [reviewed in Ref. (8)]. Other studies have defined three distinct phenotypic bovine monocyte subsets based on their variable surface expression of CD14 and CD16 among CD172a^+^ cells (40). Expression of CD11c and CD172a is considered constitutive in bovine monocyte subsets, while expression of CD11b is variable (41). We did not find significant expression of CD163 on monocytes in our study; however, expression of CD163 has been described by others in bovine monocytes (41). A possible explanation for these contradictory findings is the utilization of different clones of the CD163 monoclonal antibody. We used a murine anti-bovine clone (LND68A) while Corripio-Miyar et al. (41) used human clone (EDHu-1); potential concerns regarding interspecies cross-reactivity of monoclonal antibodies have been published (42).

In our model CD1b, CD11c, CD14, and CD205 were all upregulated following *in vitro* differentiation of monocytes; however, these particular markers do not reliably distinguish MDMs from MDDCs. Our data do suggest that MDMs can be phenotypically distinguished from MDDCs because of significantly higher expression of CD11b, CD163, and CD172a on MDMs compared to MDDCs. Bovine MDDCs have been described as CD172a^+^ while the expression of CD1b, CD11b, CD14, and CD205 vary depending on the subset of MDDC [reviewed in Ref. (8)]. MDMs have historically been classified by phenotype and function using surface-marker expression and cytokine-secretion profiles, respectively, but more recently classification of MDMs as either classically (M1) and alternatively activated (M2) macrophages using expression of CD163 has been described. M1 macrophages are CD163^−^ and secrete pro-inflammatory cytokines while M2 macrophages are CD163^+^ and secrete low levels of pro-inflammatory cytokines and high levels of IL-10 (43). In our *in vitro* model, we neither identified CD163^−^ populations of MDMs nor assessed cytokine concentration in supernatants. Thus, further research is required to characterize and classify cells of the bovine MPS under different isolation (a.k.a. magnetic beads, FACS, adherence), culture conditions *in vitro* and evaluating other relevant surface markers such as CD209 (DC-SIGN) (44), CD16 (41), CD68 (45), and CD11a (46). Regardless, our data support the basic hypothesis that MPS cells comprise a complex network of distinct cell subsets that though they share some overlapping phenotypic and functional characteristics, they polarize depending on the local microenvironment for specific functions (47).

After coculturing freshly isolated blood monocytes with sorted WC1^+^ and WC1^neg^ γδ T lymphocytes (dMonWC1^+^ and dMonWC1^neg^, respectively) during the differentiation process, our data indicate that the presence of γδ T lymphocytes has no phenotype-altering effect on monocyte differentiation. Viability of *Map* recovered from dMonWC1^neg^, however, was significantly reduced suggesting that the presence of WC1^neg^ γδ T lymphocytes improves the ability of differentiated monocytes (dMonWC1^neg^) to limit *Map* viability. In a previous study, we showed that the presence of either WC1^+^ or WC1^neg^ γδ T lymphocytes cocultured with autologous *Map*-infected MDMs from 30- to 40-day-old calves was associated with reduced viability of *Map* recovered from MDMs (26). Taking into account that (1) in the previous study, MDMs were considered fully differentiated prior to the addition of γδ T lymphocytes into cocultures, (2) in the current study, significant differences were observed between the viability of *Map* recovered from dMonWC1^+^ and dMonWC1^neg^, and (3) when *Map* was introduced in the current study, WC1^+^ and WC1^neg^ γδ T lymphocytes had been removed; we hypothesize that dMonWC1^neg^ are distinct from classical MDMs, and that WC1^neg^ γδ T lymphocytes alter the functional differentiation of peripheral blood monocytes. Based on these data, our hypothesis is that WC1^neg^ γδ T lymphocytes have a direct effect on transcription factor expression during monocyte differentiation resulting in a monocyte-derived cell with increased ability to limit *Map* viability. Because we have observed that the number of γδ T lymphocyte subsets within tissues of mucosal surfaces is variable between calves (unpublished data), we hypothesize that inter-animal variability also influences the different immune responses and disease outcomes that can be commonly observed within groups of calves (i.e., a herd with endemic *Map* infection). This inter-animal variability might explain why some animals (i.e., those with more WC1^neg^ γδ T lymphocytes or those that have more cognate γδ T lymphocyte/MPS cell interactions in the ileum) clear *Map* infection and do not progress to later stages of bovine paratuberculosis. Further studies are required to characterize the distribution and function of bovine γδ T lymphocytes in different mucosal and non-mucosal tissues.

To determine how γδ T lymphocyte subsets affect DC maturation, we first defined the characteristics of both iMDDC and mMDDC in our system by evaluating expression of specific maturation markers and co-stimulatory molecules. As expected and based on the current literature, our data confirm that MHC-I, CD80, and CD86 are useful markers to differentiate mMDDCs from iMDDCS because they are upregulated during the maturation process. Other bovine models have shown that *Salmonella typhimurium*-infected DCs had significantly increased expression of MHC-I, MHC-II, CD40, CD80, and CD86 (48). In our system, MHC-II was not upregulated on mMDDCs at 48 h post *Map* infection; however, the expression of MHC-II was highly variable between animals in our study. Inter-animal immune cell phenotypic variability has been well established in bovine studies of our lab and others (25, 26, 49), and the distinct pattern of high/low-effector functions and specific phenotypes in cattle is complex but probably explains an individual’s unique ability to respond (or not) to infection. Timing may also have influenced our ability to detect early and transient changes in MHC-II expression. It is known that after stimulation of murine DC PAMPs receptors, MHC-II expression increases transiently but then decreases (50), though this phenomenon has not been shown in bovine DCs. Furthermore, it is known that MHC-II expression on bovine MDMs is downregulated between 24 and 48 h post *Map* infection (51) and because cells in this study were analyzed at only a single time point (48 h after *Map* infection), we may have thus been unable to detect early differences in the expression of MHC-II due to timing.

The presence of WC1^neg^ γδ T lymphocytes in our coculture experiment was associated with significantly increased expression of MHC-II on iMDDC + WC1^neg^ cells compared with iMDDCs and mMDDCs. Upregulation of MHC-II, along with CD86 and CD83 has also been induced by human Vγ9Vδ2 T lymphocytes on DCs, suggesting the ability of γδ T lymphocytes to specifically promote DC maturation (17). Furthermore, human iMDDCs induce Vγ9Vδ2 T lymphocytes to secrete pro-inflammatory cytokines required for their own maturation (52). This reciprocal effect has not been definitively demonstrated in cattle, and further research is required to determine if bovine DCs could induce this effect on either WC1^+^ and/or WC1^neg^ γδ T lymphocytes.

A major functional change during MDDC maturation is reduced phagocytic capacity of MDDCs; this finding is supported by studies in adult cows (28). In our study, a reduced phagocytic capacity was observed in iMMDCs cocultured with either WC1^+^ or WC1^neg^ γδ T lymphocyte subsets, which suggests that WC1^+^ or WC1^neg^ γδ T lymphocytes induce “functional” MDDC maturation with respect to phagocytic capacity.

Our overall hypothesis was that early γδ T lymphocytes/MPS/*Map* interactions influence the initiation of early local host immunity and potentially the induction of adaptive immunity and progression or eventual outcome of *Map* infection. To test our hypothesis, we first needed to define the phenotype and effector functions of MPS cells specifically under *in vitro* conditions in our laboratory. We have shown that cells from the MPS can be distinguished by collective examination of phenotype and function: (1) monocytes lack the expression of CD1b, CD205, and CD163; (2) MDMs express higher levels of CD11b, CD163, and CD172a compared to MDDCs; (3) mMDDCs express higher levels of CD11c, CD80, and CD86 compared to iMDDC; mMDDCs have reduced phagocytic capacity compared to iMDDCs. In this study, the most significant findings related to the effect of γδ T lymphocytes include: (1) the presence of WC1^neg^ γδ T lymphocytes contributes to differentiation of monocytes into cells with increased ability to limit *Map* viability and (2) both γδ T lymphocyte subsets induce functional MDDC maturation (reduced phagocytosis).

## Ethics Statement

All animal procedures in this study were approved by the Institutional Committee on Animal Care at the University of Guelph (Animal Utilization Protocol # 3373).

## Author Contributions

MMB and BLP performed the experiments; designed the experiments; interpreted the data; drafted the manuscript; reviewed and approved the final version of the manuscript; agreed to be accountable for the content of the work.

## Conflict of Interest Statement

The authors declare that the research was conducted in the absence of any commercial or financial relationships that could be construed as a potential conflict of interest.
