# Supplementary material for: Bovine WC1+ and WC1neg γδ T Lymphocytes Influence Monocyte Differentiation and Monocyte-Derived Dendritic Cell Maturation during In Vitro Mycobacterium avium Subspecies paratuberculosis Infection
Source: Front Immunol. 2017 May 22;8:534. doi: 10.3389/fimmu.2017.00534 (PMC5439176; doi:10.3389/fimmu.2017.00534)
Supplement: Supplementary file 1 [file Data_Sheet_1.PDF]

Supplementary Material

Bovine WC1^{+} and WC1^{neg} γδ T lymphocytes influence monocyte differentiation and monocyte-derived dendritic cell maturation during *in vitro Mycobacterium avium* subspecies *paratuberculosis* infection

Monica M. Baquero*, Brandon L. Plattner

*** Correspondence:** Monica M. Baquero: mbaquero@uoguelph.ca

1 Supplementary Data: Phenotypic characterization of cells from the MPS.

1 x 10^{6} sorted WC1^{+} or WC1^{neg} γδ T lymphocytes were added directly to wells containing 2 x 10^{5} monocytes the same day of PBMC isolation (day 0). After 6 days, cultures of MDMs, iMDDC, monocytes differentiated in presence of WC1^{+} (dMonWC1^{+}) or WC1^{neg} (dMonWC1^{neg}) γδ T lymphocytes were obtained. On day 6, MDMs, iMDDC, dMonWC1^{+}, dMonWC1^{neg} or iMDDCs were infected at a multiplicity of infection (MOI) of 10:1 for 48 hours with an Ontario-derived clinical bovine *Map* strain (gc86). Expression of CD1b, CD11c, CD14, CD205, CD11b, CD163 and CD172a on bovine uninfected monocytes, MDMs, iMDDCs, mMDDCs, dMonWC1^{+} and dMonWC1^{neg}; and *Map*-infected uninfected MDMs, iMDDCs, dMonWC1^{+} and dMonWC1^{neg} was assessed using flow cytometry.

Table S1. ***Mean and standard deviation (SD) of CD1b, CD11c, CD14, CD205, CD11b, CD163 and CD172a on bovine uninfected monocytes, MDMs, iMDDCs, mMDDCs, dMonWC1+ and dMonWC1neg; and Map-infected uninfected MDMs, iMDDCs, dMonWC1+ and dMonWC1neg.***

Cell	*Map*	Statistic	CD1b	CD11c	CD14	CD205	CD11b	CD163	CD172a
Monocytes	-	Mean	69.28	4489.00	5735.14	663.00	15577.29	213.57	7260.71
		SEM	10.67	581.97	499.27	69.48	988.42	32.99	1213.25
MDMs	-	Mean	1323.00	15416.14	37206.43	16503.14	110632.00	2627.57	40690.71
		SEM	106.99	3005.71	3664.81	7852.11	5921.22	572.11	5412.30
	+	Mean	1284.00	13863.29	42626.86	14324.29	115421.14	4188.57	40342.71
		SEM	95.92	1540.27	5215.46	6680.39	4814.98	1353.97	4564.69
iMDDCs	-	Mean	1108.14	16485.14	45349.71	11145.29	68347.57	1439.71	28352.14
		SEM	198.19	3767.77	13571.81	5552.68	7789.49	343.98	3285.51
	+	Mean	1047.57	20245.57	38393.71	8325.14	63025.29	2000.71	25230.14
		SEM	164.71	5847.51	16046.15	3726.88	8062.27	555.87	2232.43
mMDDCs	-	Mean	1151.00	24375.86	64507.00	9792.57	55578.43	2284.14	31451.57
		SEM	135.81	4739.74	28706.83	4028.30	2793.20	332.62	2056.55

dMonWC1^{+}	-	Mean	1628.43	20623.57	43184.86	14143.14	111670.14	2488.29	38105.72
		SEM	147.92	2381.99	4905.30	5602.74	6003.64	411.07	3853.64
	+	Mean	1619.14	21168.86	47092.43	16030.29	100165.29	2574.29	41115.43
		SEM	111.17	1640.02	4642.45	7697.03	6465.19	622.98	4964.18
dMonWC1^{neg}	-	Mean	1518.57	14133.86	44566.71	14721.00	105290.14	2521.29	38180.29
		SEM	131.10	1563.93	4802.69	6506.84	8628.55	463.43	4876.05
	+	Mean	1540.71	14732.29	41207.86	18052.57	96351.57	2812.00	41153.00
		SEM	96.51	1874.07	3359.93	7741.25	4892.48	584.61	5090.11

2 Supplementary Data: Flow cytometry gating strategy

During flow cytometric analysis, flow stability was assessed to ensure a good and even flow stream during the instrument's run. Then the region of interest was identified using a forward and side scatter (SSC-A Vs. FSC-A) gating to identify the cells of interest based on the relative size and complexity of the cells, while removing debris and other events that are not of interest. The pulse geometry gating was done to remove doublets from the dataset (FSC-A Vs. FSC-H). A cell viability dye (Zombie NIR) was used to remove from the analysis dead cells which can non-specifically take up antibodies. Finally, data analysis controls included gating controls (unstained cells, single stained fluorescent cells and fluorescent minus one (FMO) controls to strategically place gates to clearly discriminate positive from negative cells.

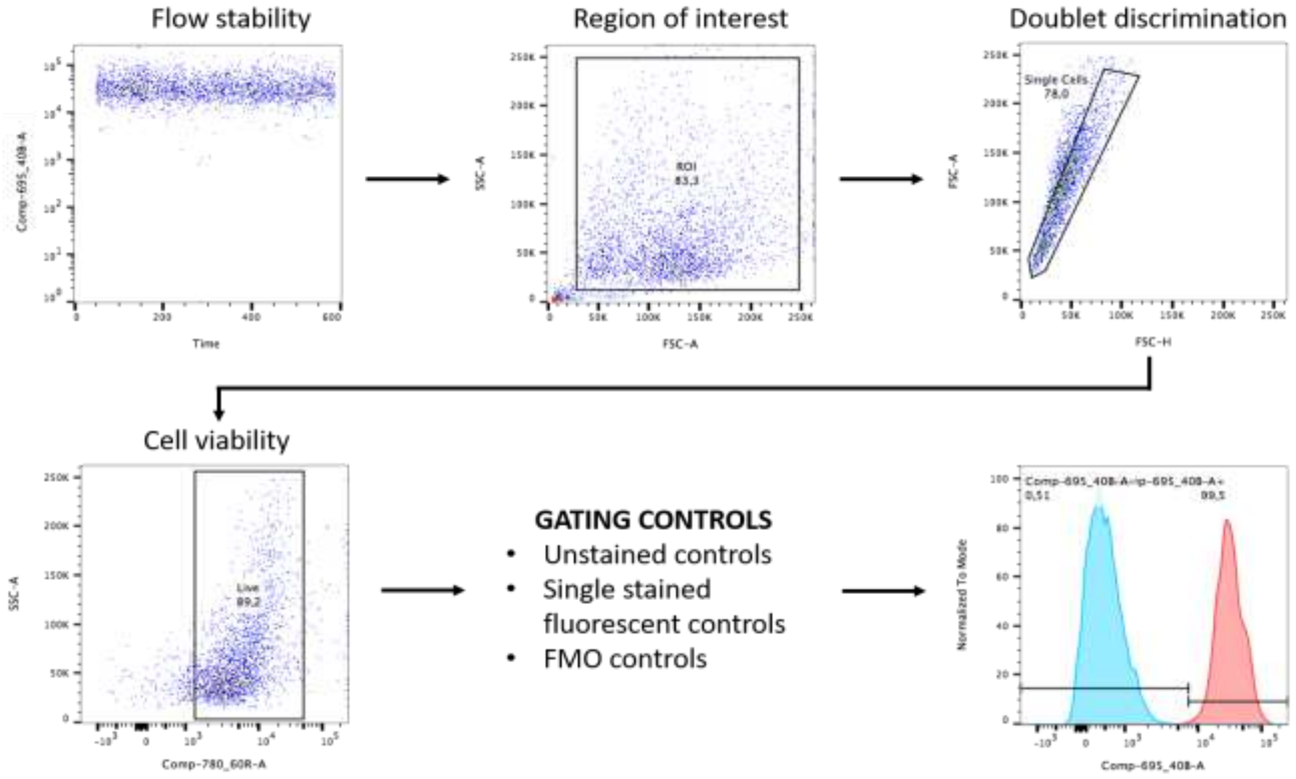

Figure S2. Flow cytometry gating strategy
